# Anxiety among students during the pandemic - Results from the C-19 German Student Well-being Study: Eileen Heumann

**DOI:** 10.1093/eurpub/ckac131.478

**Published:** 2022-10-25

**Authors:** E Heumann, SM Helmer, H Busse, S Negash, CR Pischke, J Trümmler, Y Niephaus, C Stock

**Affiliations:** 1 Institute for Health and Nursing Science, Charité - Universitätsmedizin Berlin, Berlin, Germany; 2 Human and Health Sciences, University of Bremen, Bremen, Germany; 3 Department Prevention and Evaluation, Institute for Prevention Research and Epidemiology, Bremen, Germany; 4 Institute for Medical Epidemiology, Martin Luther University Halle-Wittenberg, Halle (Saale), Germany; 5 Institute of Medical Sociology, Heinrich Heine University Duesseldorf, Duesseldorf, Germany; 6 Department of Social Sciences, University of Siegen, Siegen, Germany

## Abstract

**Background:**

Anxiety is widespread among university students. The COVID-19 pandemic af-fected students’ mental health negatively. Given the long duration of the pandemic monitoring mental health remains important. This study aims to determine to which extent anxiety is preva-lent among students (1), what factors are associated with it (2) and which student groups are mostly affected (3).

**Methods:**

The cross-sectional COVID-19 German Student Well-being Study (C19 GSWS) sur-veyed mental health and well-being of students at five universities in Germany from 27.10.-14.11.21. Anxiety was assessed using the GAD-2. Associations between anxiety and sociodem-ographic, socioeconomic/social support factors as well as health- and COVID-19-related factors were determined using multiple binary logistic regression models.

**Results:**

The mean age of students was 24.1 years (SD = 4.9), 67% were women and 31% men. The prevalence of anxiety was 32% and diverse gender (OR = 3.98, 95% CI: 1.71-9.23), a com-plicated relationship status (OR = 1.66, 95% CI: 1.06-2.60), the lack of a confidant (OR = 2.50, 95% CI: 1.80-3.46), and financial difficulties (e.g., being able to cover monthly expenses; OR = 1.76, 95% CI: 1.36-2.29) were associated with anxiety. Participants who were worried about (re)infection with COVID-19 had a 1.28-times higher chance (OR, 95% CI: 1.03-1.59) for anxie-ty. Students who were (rather) not worried that a relative would become severely ill with COVID-19 had a lower chance to experience anxiety (OR = 0.72, 95% CI: 0.53-0.98) as well as those who were confident receiving medical care in case of an infection with COVID-19 (OR = 0.80, 95% CI: 0.65-0.98).

**Conclusions:**

Concepts for prevention and counselling in terms of mental health problems in students should be developed considering specific stressors due to the pandemic.

**Key messages:**

• This study shows that anxiety is widespread among university students and associated with a variety of stressors.

• The findings can help to develop specific concepts for prevention and counselling.

